# CAM Models: Lessons and Implications for CAM Evolution

**DOI:** 10.3389/fpls.2022.893095

**Published:** 2022-06-23

**Authors:** Asdrubal Burgos, Enoc Miranda, Ester Vilaprinyo, Iván David Meza-Canales, Rui Alves

**Affiliations:** ^1^Laboratorio de Biotecnología, CUCBA, Universidad de Guadalajara, Guadalajara, Mexico; ^2^Institute of Biomedical Research of Lleida, IRBLleida, Lleida, Spain; ^3^Departament de Ciències Mèdiques Bàsiques, Universitat de Lleida, Lleida, Spain; ^4^Departamento de Ecología Aplicada, CUCBA, Universidad de Guadalajara, Guadalajara, Mexico; ^5^Unidad de Biología Molecular, Genómica y Proteómica, ITRANS-CUCEI, Universidad de Guadalajara, Guadalajara, Mexico

**Keywords:** CAM, CAM evolution, ODE models, FBA models, carbon concentration mechanism

## Abstract

The evolution of Crassulacean acid metabolism (CAM) by plants has been one of the most successful strategies in response to aridity. On the onset of climate change, expanding the use of water efficient crops and engineering higher water use efficiency into C3 and C4 crops constitute a plausible solution for the problems of agriculture in hotter and drier environments. A firm understanding of CAM is thus crucial for the development of agricultural responses to climate change. Computational models on CAM can contribute significantly to this understanding. Two types of models have been used so far. Early CAM models based on ordinary differential equations (ODE) reproduced the typical diel CAM features with a minimal set of components and investigated endogenous day/night rhythmicity. This line of research brought to light the preponderant role of vacuolar malate accumulation in diel rhythms. A second wave of CAM models used flux balance analysis (FBA) to better understand the role of CO_2_ uptake in flux distribution. They showed that flux distributions resembling CAM metabolism emerge upon constraining CO_2_ uptake by the system. We discuss the evolutionary implications of this and also how CAM components from unrelated pathways could have integrated along evolution.

## Introduction

Crassulacean acid metabolism is a photosynthetic adaptation syndrome of carbon concentration that has evolved multiple times in plants (Crayn et al., 2004; Winter and Smith, 2012). It consists in the nocturnal fixation of CO_2_ into malate, which is stored in the vacuole and decarboxylated the next day providing CO_2_ for Rubisco (Dodd et al., 2002). It is commonly associated with conditions of water scarcity in which plants close their stomata during the day to avoid water loss. Keeping stomata closed during the day while concentrating CO_2_ at night permits a high water use efficiency, allowing plants to remain productive even under dry conditions. A great interest in CAM crops has recently developed, given the potential of extending their use in semi-arid and degraded lands (Liu et al., 2018). In natural ecosystems, CAM plants are less affected by increased nighttime temperatures and rainfall variability than C4 grasses (Huang et al., 2020) and are increasing their cover in many arid regions (Yu and D’Odorico, 2015). Global climate models predict drier regimes and decrease in soil moisture in large areas of the world (Dai, 2013). In many of today’s agricultural lands, CAM crops could be the only crops able to grow in a fairly close future. Understanding CAM evolution is especially relevant for CAM engineering into C3 crops. The multiple occurrences of CAM in numerous lineages across the plant kingdom and different levels of CAM expression suggest that the evolutionary path from C3 to CAM is relatively straightforward (Schiller and Bräutigam, 2021). Finding the key events that led to CAM emergence can potentially pinpoint the key transformations necessary for engineering CAM into C3 plants.

Mathematical models on CAM metabolism provide valuable information. Two types of models have been used to study CAM: ordinary differential equations (ODE) and flux balance analysis (FBA) models. ODE-based models can accurately describe metabolite concentrations and their change over time at the cost of requiring quantitative knowledge on the parameters and enzyme activities of the pathways being modeled. On the other hand, FBA-based models require no quantitative *a priori* knowledge. They can be used to study steady state flux distributions but provide no information on concentrations or transient dynamics.

ODE-based models have determined the components and regulatory interactions sufficient to bring about CAM behavior, while FBA models have shown that many CAM defining features —such as nocturnal malate accumulation—are the result of flux optimization upon limited CO_2_. Here, we review the findings of both groups of models. Although most of the works were not conceived with evolution in mind, several of their findings have direct evolutionary implications, in particular for the ongoing discussion on the C3-to-CAM evolutionary continuum (Cushman and Bohnert, 1999; Dodd et al., 2002; Silvera et al., 2010; Bräutigam et al., 2017).

## CAM Biochemistry

Crassulacean acid metabolism is divided into four archetypical phases. Phase I occurs at night when the stomata are open and starch reserves are used to synthesize phosphoenolpyruvate (PEP). High amounts of PEP are needed to fix CO_2_ overnight, a reaction catalyzed by PEP carboxylase (PEPC). PEP carboxylase is reversibly phosphorylated during the night to reduce malate feedback inhibition (Carter et al., 1991). Oxaloacetate (OAA) is the product of PEPC and is quickly converted by malate dehydrogenase into malate (Cuevas and Podestá, 2000) which is transported into the vacuole. A vacuolar H^+^-ATPase moves large quantities of H^+^ into the vacuole generating a proton gradient that energizes malate transport (Cheffings et al., 1997). Phase II occurs during the first hours of dawn. In this phase, the transition from PEPC-mediated to Rubisco-mediated CO_2_ fixation occurs, and for a period of time CO_2_ is fixed by both carboxylases (Lüttge, 2015). Phase III takes place during the day while stomata are closed. During this phase, CO_2_ is freed from malate and re-fixed by Rubisco. For this to happen, malic acid is re-mobilized by passive efflux and decarboxylated. There are two alternative pathways for malate decarboxylation, depending on the species. One starts with the release of CO_2_ by the conversion of malate to pyruvate by NAD(P)-ME, followed by the regeneration of PEP by PPDK (Kondo et al., 2000). In a second pathway, NAD(P)-MDH converts malate to OAA, followed by PEPCK, regenerating PEP, and releasing CO_2_ (Cushman and Bohnert, 1999). Finally, phase IV takes place late in the afternoon once malate reserves have been depleted. At this time, stomata open again and allow for the uptake of CO_2_, which is processed directly by Rubisco (Dodd et al., 2002).

The occurrence of CAM four phases varies from species to species and depends on the developmental stage or the severity of environmental stress. Young tissues of all CAM plants perform C3 photosynthesis (Winter, 2019). Upon drought stress, phases II and IV can be lost or reduced in constitutive CAM plants (Dodd et al., 2002). The proportion of PEPC-fixed CO_2_ also increases with drought in species with a C3-CAM intermediate metabolism, while facultative CAM species can revert completely to C3 after rewatering (Winter and Holtum, 2014). In addition, several CAM modes are possible; including CAM cycling, consisting in respiratory CO_2_ re-fixation, but with stomatal opening during the day; archetypical CAM; and CAM idling, which happens upon severe drought, in which only respiratory CO_2_ is re-fixed and stomata remain closed day and night (Borland et al., 2011).

## ODE Models and the Origin of CAM Minimal Set of Components

The functioning of CAM metabolism results in the diurnal oscillation of malate content and CO_2_ uptake. As the oscillations continue despite leaving the plants in continuous light, or darkness and CO_2_-free air (Wilkins, 1959, 1960; Nuernbergk, 1960), it became clear that understanding these dynamics would require a mathematical framework. Nungesser et al. (1984) translated a basic scheme of reactions known to be relevant for CAM metabolism into a set of ordinary differential equations (ODE). This so-called skeleton model would become the basis for a series of models that would continue for two more decades, dealing with the perturbation of the rhythmicity as well as with finding the dynamically essential components (Table 1).

**Table 1:** Distinctive features of ODE-based models addressing CAM diel rhythmicity.

Model	Modified from	Focus	Modifications from previous models	Main achievements
Nungesser et al., 1984	–	Interaction between light and metabolite pools.	–	Reproduced CAM behavioral parameters such as content of malic acid, starch, Glc6P and PEP, CO_2_-exchange, and C_i_.
Lüttge and Beck, 1992	Nungesser et al., 1984	CAM rhythmicity, light and temperature.	Removed the influence of light on malate transport. Allowed light fluxes to vary arbitrarily. Adjusted parameters to stabilize oscillations for runs longer than a day/night cycle.	Reproduced a stable rhythmicity in normal dark–light cycles and in continuous light. Predicted accurately a change to chaos as irradiance and temperature is increased.
Grams et al., 1996	Lüttge and Beck, 1992	Effect of irradiance and temperature on CAM rhythmicity.	Included the saturation of CO_2_ fixation at high irradiance and high C_i_. Included three different modes of modeling malate transport: influx, efflux, and influx near maximum capacity.	Predicted accurately that high irradiances gradually make oscillations disappear and that rhythm displays a smaller amplitude upon re-initiation. Reproduced the effect of below-range temperature halting rhythmicity.
Grams et al., 1997	Grams et al., 1996	Low and high temperature effect on CAM rhythmicity.	Influx, efflux and influx near maximum capacity were modeled as a function of temperature.	Reproduced accurately the phase displacement upon re-initiation of CAM rhythmicity after out-of-range temperature treatments.
Blasius et al., 1997	Lüttge and Beck, 1992; Grams et al., 1996	Effects of temperature as a continuous functional dependency.	Reduced to four the number of metabolite pools modeled. Added an algorithm to simulate continuous temperature variations.	The model exhibits robustness against functional changes in its structure. Increases in light intensity under continuous light increased the oscillation frequency but did not disrupt the rhythmicity. Higher temperatures resulted in slower oscillations.
Blasius et al., 1998	Blasius et al., 1997	Constructing a minimal skeleton model.	Removed the starch pool Added a respiration term into the analysis Adjusted the ratio of cytoplasm/vacuole volume to match an experimentally determined value.	Showed that only malate in the vacuole, malate in the cytoplasm and C_i_ in the earlier model are dynamically independent Showed that PEPC phosphorylation cannot sustain CAM rhythmicity on its own Provided evidence that CAM rhythmicity relies on a hysteresis switch at the tonoplast.
Blasius et al., 1999	Blasius et al., 1998	Implementation of a continuous hysteresis switch	Included a dynamic switch, using an equation that simulates membrane dynamics.	The appearance of unstable steady states allows the system to reproduce more closely experimental data More accurate simulation of phase I.
Wyka et al., 2004	Blasius et al., 1999	Testing the effect of removing ambient CO_2_	Tested both experimentally and computationally different durations and moments for CO_2_ removal.	The model did not reproduce experimental results. Simulating CO_2_ removal for a time period led to a phase shift in oscillations, while *in vivo* the oscillations kept as normal.
Owen and Griffiths, 2013	Nungesser et al., 1984; Blasius et al., 1999	Identifying key flow junctions, metabolic feedbacks and parameters that limit CO_2_ uptake over the diel cycle.	Implemented a system dynamics approach. Included a wider set of elements, regulatory interactions in the model and parameters, including carbonic anhydrase reaction, the switch between PEPC and Rubisco. carboxylation, transpiration and mesophyll conductance.	Modifying a number of parameters including vacuolar capacity, stomatal and mesophyll conductance as well as switches from PEPC to Rubisco activity allowed the initial model fitted to *K. draigemontiana* to replicate *Agave tequilana* behavior in terms of CO_2_ uptake.

**Figure 1:**
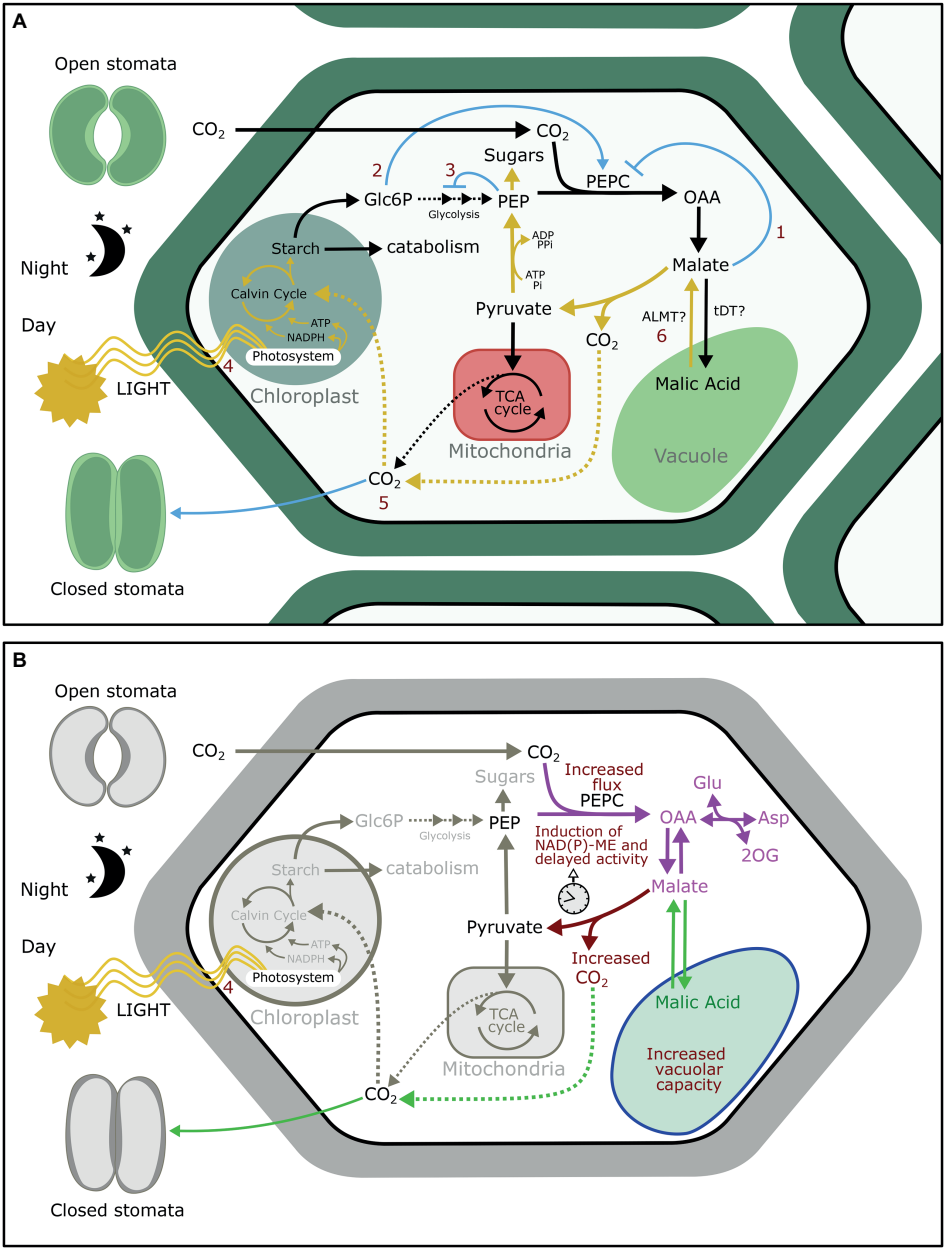
**(A)** Regulatory interactions sufficient to give rise to diel oscillations of malate accumulation and CO_2_ uptake adapted from Nungesser et al. (1984) skeleton model: malate inhibition of PEPC (1); Glc6P activation of PEPC (2); PEP inhibition of glycolysis (3); light activation of photosynthesis (4); induction of stomatal closure by high C_i_ (5); and the autonomous vacuolar oscillator (6). **(B)** Pathways that articulated during CAM evolution. The anapleurotic fixation of CO_2_ by PEPC in unicellular algae occurs to provide carbon skeletons for amino acid synthesis (magenta). The same pathway is found in C3 plants, which store in the vacuole malic acid resulting from CO_2_ fixation during the night to be used for amino acid synthesis the next day. C3 plants are also able to close stomata upon high C_i_ (green). Three events could have closed the cycle to give rise to CAM (brown): an increased flux through PEPC; an increase in decarboxylation activity, e.g., NAD(P)-ME, and its delay to not overlap with PEPC fixation; and finally, an increased vacuolar capacity that allows the concentration of CO_2_.

Nungesser et al. (1984) skeleton model included six regulatory steps: inhibition of PEPCase by malate, activation of this last reaction by Glc6P, inhibition of starch consumption by PEP, activation of photosynthesis by light intensity, inhibition of CO_2_ uptake by increased internal concentration of CO_2_ (C_i_), and activation of malate transport from the vacuole to the cytosol by light (Figure 1). The predicted relative content of Glc6P, PEP, malic acid, starch, C_i_, and net CO_2_ exchange along the 24 h cycle were remarkably in agreement with experimental data. This shows that the enzymes and metabolite pools considered in the model are sufficient to explain the basic features of CAM.

Nungesser et al. (1984) assumed that the transport of malate between the vacuole and the cytoplasm was stimulated by light. However, oscillations in malate content under continuous light challenged this idea. To solve this issue, they added an oscillating element—a hysteresis switch—controlling malate transport through the tonoplast that would be used in the future by other authors. Lüttge and Beck (1992) refined the model by adjusting several parameters to stabilize oscillations under prolonged runs, kept the hysteresis switch, and eliminated the direct influence of light on malate transport. The changes allowed the simulations to reproduce the endogenous malate oscillations under continuous light.

Three subsequent refinements improved model fitting to the observed effect of irradiance and temperature on oscillations (Blasius et al., 1997, 1998, 1999). The original six pools of metabolites were reduced to only three: cytoplasmic and vacuolar malate pools, and C_i_. This model assumes an active influx filling the vacuole with malate during the night. At a critical concentration, the hysteresis switch kicks in and vacuolar malate is rapidly expelled. C_i_ is the carbon source for malate production, while malate consumption and CO_2_ assimilation connect the oscillatory behavior with the rest of metabolism. Blasius et al. (1999) improved the mechanistic details that generated the hysteresis switch by considering membrane properties and simulating tonoplast permeability as a function of membrane order.

The precise malate transporter responsible for the hysteresis switch remains to be identified. Meyer et al. (2011) found that malate channel ALMT6 shifts from inward to outward rectification in Arabidopsis guard cells at a low vacuolar pH. It was recently suggested that a similar mechanism could operate in CAM plants (Ceusters et al., 2021). Thus, it might be a drop in pH and not a critical vacuolar turgor that triggers the influx-to-efflux shift to begin malate decarboxylation. Transcriptomics data from the CAM species *Agave americana* suggest the malate/citrate antiporter tDT might also play a role in overnight malate accumulation (Yin et al., 2018).

The fact that skeleton models are sufficient to reproduce CAM dynamics suggests that CAM can originate from the emergence of such metabolic circuits during evolution. After a quick literature survey based on the model by Nungesser et al. (1984), we found evidence that not only enzymes but also regulatory interactions are already in place in C3 plants (Figure 1). These regulatory interactions play roles in different primary metabolism pathways and date back to different stages in the evolution of life. PEP inhibition of glycolytic enzymes is a feedback regulation mechanism that is shared with heterotrophic bacteria and animals (Plaxton, 1996; Ogawa et al., 2007; Grüning et al., 2014). Malate inhibition of PEPC has been documented in unicellular green algae and is crucial for coordinating carbon metabolism with nitrogen assimilation (Peak and Peak, 1981; Schuller et al., 1990). The activation of photosynthesis by light and the subsequent carbon fixation by Rubisco is common for all photosynthetic organisms (McKay et al., 1991; Portis et al., 2008; Tsai et al., 2015). Stomatal closure induction by high C_i_ is known to occur in early vascular plants and is thought to have been essential for the colonization of land by plants (Chater et al., 2011). Glc6P activation of PEPC is found in C3, C4, and CAM plants (Chollet et al., 1996; Blonde and Plaxton, 2003). In C3 plants, this regulation plays a role in gluconeogenesis by regulating the rate of starch mobilization with the production of dicarboxylic acids (Law and Plaxton, 1995). Finally, there is evidence that the autonomous vacuolar oscillator is also present in C3 plants. As malate can be toxic for the cell in large quantities and is essential for a number of biochemical pathways, its amount in the cytosol is tightly regulated by storing excess malate in the vacuole (Fernie and Martinoia, 2009). Vacuoles of C3 plants switch from malate influx to efflux depending on a critical concentration of cytosolic malate (Gout et al., 1993). As in CAM plants, malate in the vacuole of C3 plants fluctuates diurnally (Gerhardt et al., 1987).

## FBA Models Recreate CAM by Constraining C3 Metabolism

The accumulated information on biochemical reactions together with the availability of genomic data made the first FBA genome-wide metabolic models possible (Schilling and Palsson, 1998; Schilling et al., 1999; Edwards and Palsson, 2000). In plants, the first FBA whole-genome (FBA-WG) metabolic reconstruction was done for Arabidopsis (Poolman et al., 2009), followed by maize (Saha et al., 2011). Later, Cheung et al. (2013) incorporated transport and maintenance costs into the Arabidopsis whole-genome FBA model, allowing for a more realistic prediction of flux distribution in central metabolism. Building on this improvement, Cheung et al. (2014) incorporated the interaction between diurnal and nocturnal metabolism (Table 2). Essentially, this was done by creating two alternative models corresponding to each phase. The models were coupled by adding “transport” reactions between them that would pass on accumulated metabolites. A specified amount of sucrose and amino acids was set to be exported to the phloem, thus requiring the cell to be productive. The flux distribution of both models was simultaneously optimized to decrease the overall amount of material flowing through metabolism. Since the study addressed diel metabolic changes, it was natural to use the model to compare C3 and CAM scenarios. This was done by setting CO_2_ exchange to zero during the night for CAM. Remarkably, the simulations are in agreement with knowledge on plant metabolism. The C3 simulation predicted citrate to accumulate during the night and to be used during the day for amino acid biosynthesis. For CAM, the nocturnal accumulation of malate was predicted with PEPC as the carboxylating enzyme.

**Table 2:** Distinctive features of FBA-based models addressing carbon flux re-distribution in CAM.

Model	Modified from	Focus	Modifications from previous models	Additional constraints
Cheung et al., 2014	Cheung et al., 2013	Interaction between day and night phase of metabolism	Coupled two identical genome-wide models representing either day or night and modeled as a single optimization problem. Implemented different constraints at each phase. Each phase was given the choice of accumulating sucrose and amino acids. Compared C3 with CAM scenarios	No CO_2_ uptake during the night for the CAM scenario. Ratio of sucrose and amino acid export to the phloem: 3:1 (day:night) Ratio of nitrogen import from the xylem into the leaf: 3:2 (day:night) Amino acids could only accumulate during the day
Shameer et al., 2018	Cheung et al., 2014	Calculating energetic costs for C3 and CAM	Reduced the model to a core stoichiometric model of central plant metabolism (641 reactions and 555 metabolites) Considered explicitly organellar pH and charge state of metabolites	Phloem export according to tomato phloem sap content for the C3 scenario. Phloem export according to *O. ficus-indica* phloem sap content for the CAM scenario.
Töpfer et al., 2020	Shameer et al., 2018	CAM interaction with the environment	Divided the two-phase diel model into a 24-phase model. Added a simplified gas diffusion to test the model under measured relative humidity and temperature Tested different malate storage capacities. Implemented Pareto frontier analysis to evaluate CO_2_ demand and water saving tradeoff	Unconstrained CO_2_ uptake for both scenarios C3 average vacuolar capacity for C3 scenario *K. daigremontiana* vacuolar capacity or unrestricted vacuolar capacity for CAM scenario
Tay et al., 2021	Shameer et al., 2018	CAM cycling, CAM idling and C3-CAM evolution	Considered that O_2_ could accumulate and be transferred to the next phase. Made a series of models gradually decreasing gas exchange	Phloem export, O_2_ and CO_2_ exchange set to zero for CAM idling scenario. O_2_ and CO_2_ exchange set to zero during the day phase for CAM cycling scenario. O_2_ and CO_2_ exchange constrained to different values between 13.12 and 0 μM m^−2^ s^−1^ for the series with decreased gas exchange

Shameer et al. (2018) simplified Cheung’s FBA-WG model (Cheung et al., 2014) to a core stoichiometric model of central plant metabolism and considered explicitly organellar pH and the charged state of metabolites. The modifications allowed the improved model to match experimental measurements of starch and malate and revealed that CAM metabolism is more productive—higher phloem export rates—than C3, compensating the higher energetic costs of CAM metabolism. These results are highly valuable as a long-sought goal of biotechnologists has been the implementation of CAM into C3 crops by means of genetic engineering.

Shameer et al. (2018) model was the basis for two further works (Töpfer et al., 2020; Tay et al., 2021). Töpfer et al. (2020) extended the model by allowing for differences in vacuolar storage capacity, making gas exchange dependent on temperature and relative humidity, and considering the hourly progression of metabolism during a day. These last two additions allowed for CO_2_ uptake variations that resulted from a compromise between optimizing CO_2_, metabolic demand, and minimizing water loss. Interestingly, under a C3-like vacuole size restriction, flux optimization leads to reduced CO_2_ uptake during the hottest hours of the day—equivalent to midday depression of photosynthesis. A similar simulation under a CAM-like vacuole size restriction increased CO_2_ uptake at the end of the night together with carboxylic acid accumulation.

Tay et al. (2021) used the Shameer et al. (2018) model to study the C3-to-CAM continuum. They simulated CAM idling and CAM cycling—both considered as weak CAM—by impeding CO_2_ uptake during the night and during the whole cycle, respectively. The modified models predicted flux distribution intermediate between C3 and CAM metabolism in terms of malate and starch accumulation. In a second analysis, the authors performed a series of simulations decreasing CO_2_ uptake gradually. They observed a steady shift from C3 to CAM photosynthesis with a respective increase in flux through the starch/malate cycle.

## Discussion

Adaptive evolution reshapes metabolism to maximize fitness (Long and Antoniewicz, 2018). If we accept this, given adequately identified optimality criteria, mathematical models can be useful to understand and predict the course of evolution (Bordbar et al., 2014; de Visser and Krug, 2014). FBA models maximize fitness theoretically by translating a selective pressure into an objective function and have provided remarkably accurate predictions (Varma and Palsson, 1994; Harcombe et al., 2013). The set of FBA models studying the metabolic interactions between day and night suggests that simulating longer periods of stomatal closure are enough to trigger a flux distribution typical for CAM metabolism. Remarkably, CAM-like fluxes emerge upon constraining CO_2_ uptake on models derived from a C3 whole-genome metabolic reconstruction. Regardless of whether stomatal closure happens during the day or the night and regardless if the stomatal closure is full or partial, flux distributions resembling CAM metabolism are evident. Therefore, assuming that evolution selects for efficient responses to selective pressures (Harcombe et al., 2013), the models provide formal evidence for limited CO_2_ as the main driver of CAM evolution, a hypothesis long proposed and widely accepted (Lüttge, 2002).

Reduced CO_2_ uptake can result from limited water availability, as CAM-like fluxes appear when CO_2_ uptake is compromised by maximizing water saving (Töpfer et al., 2020). At low air humidity or when roots experience water deficit, different signals such as jasmonates and ABA induce stomatal closure (Bauer et al., 2013; De-Ollas et al., 2018). At hot dry hours, C3 and C4 plants close their stomata and undergo what is called midday depression of photosynthesis (Correia et al., 1990; Hirasawa and Hsiao, 1999). Töpfer et al. (2020) model was able to recapitulate this phenomenon. When water saving was maximized, a prolonged stomatal closure during the day was predicted. It is possible that as plants colonized dry areas or whole regions of the world became arid, the midday depression of photosynthesis widened, narrowing the time period at which CO_2_ exchange could occur. Extended hours behind closed stomata could have exerted a significant selective pressure to develop CAM so that plants could remain productive even under limited CO_2_ exchange.

A reduction in atmospheric CO_2_ has also been proposed to trigger CAM evolution, as CAM radiation events coincide with declining CO_2_ in the geological records (Keeley and Rundel, 2003; Silvera et al., 2009). Tay et al. (2021) tested this hypothesis by gradually decreasing the simulated concentration of CO_2_ and observed CAM flux distributions as well. A sustained condition of limited CO_2_ could have selected for more efficient metabolic fluxes over time that resulted in what we know as CAM. Tay et al. (2021) model provided several predictions for the metabolic transitions during CAM evolution worth testing experimentally by further works.

Although conceived with a different purpose, ODE models addressing CAM make also a significant contribution to the understanding of CAM evolution. Our quick literature survey found that not only the enzymes, but the regulatory interactions in CAM skeleton models are already in place in C3 plants. These regulatory interactions date back to different times in evolution and belong to different pathways in primary metabolism. Borrowing existing reactions is commonplace in the evolution of new pathways since it is faster and energetically less expensive than generating *de novo* reactions. The Krebs cycle is believed to have arisen from primitive pathways for amino acid synthesis (Meléndez-Hevia et al., 1996). Interestingly, CAM has also been proposed to be derived from an amino acid synthesis pathway (Bräutigam et al., 2017). In C3 plants, the accumulation of organic acids during the night fuels amino acid synthesis the following day (Gauthier et al., 2010). In this pathway, PEPC carboxylase is active during the night and catalyzes the synthesis of PEP resulting from carbohydrate degradation (Nimmo, 2003). In agreement with this, interactions (1), (2), and (3) of CAM skeleton model (Figure 1) can coordinate carbon flux toward amino acid synthesis in C3 plants: Glc6P is a feedforward activator of PEPC, while malate and PEP are feedback inhibitors of PEPC and glycolysis, respectively. Excluding vacuolar storage, this pathway is also found in unicellular green algae, in which PEPC activity is regulated to coordinate carbon metabolism with nitrogen assimilation (Peak and Peak, 1981).

The crucial step that diverts the pathway from amino acid synthesis to a new type of photosynthetic metabolism is malate decarboxylation. Malate decarboxylation connects the pathway regulated by interactions (1), (2), and (3) with carbon re-fixation by Rubisco during the day (4) and the accumulation of CO_2_ that induces stomatal closure (5; Figure 1). Either NAD(P)-ME or PEPCK are responsible for the release of CO_2_, depending on the pathway. Neither enzyme was originally part of photosynthetic metabolism (Sanwal and Smando, 1969; Boles et al., 1998; Drincovich et al., 2001), but we can presume that mutations that led to the induction of NAD(P)-ME and PEPCK activity during the day could have been evolutionarily advantageous by alleviating CO_2_ starvation during extended periods of stomatal closure. Both enzymes have been recruited multiple times during evolution when CO_2_ is limiting (Reiskind and Bowes, 1991; Magnin et al., 1997; Casati et al., 2000; Hibberd and Quick, 2002), besides their well-known role in C4 and CAM metabolism (Bovdilova et al., 2019; Lim et al., 2019). For the CAM subtypes using NAD(P)-ME, NAD(P)-ME induction could have been a direct consequence of an initial increase in PEPC activity. In heterotrophic tissues relying primarily on oxidation, PEPC and NAD(P)-ME work in concert to provide reducing power in the form of NAD(P)H (O'Leary et al., 2011). Transgenic C3 plants overexpressing PEPC exhibit an important increase in NAD(P)-ME expression and NAD(P)-ME enzymatic activity, probably triggered by an excess of cytosolic malic acid (Häusler et al., 2001). It has been previously proposed that during CAM evolution NAD(P)-ME and PEPC activities became coordinated to avoid futile CO_2_ cycling between both enzymes (Dodd et al., 2002). If high cytosolic malic acid induces NAD(P)-ME, the storage of malate during the night and the switch from malate influx to efflux in the vacuole during the day could theoretically sort out the issue of the overlap of both enzymatic activities, although this possibility has not been tested yet.

In order for CAM skeleton models to run, enough amounts of malate need to accumulate in the vacuole so that decarboxylation builds up a high C_i_ that induces stomatal closure, and thus, the reverse stomatal patterns appear. Vacuolar size is critical for CAM function (Griffiths et al., 2008). Considering this parameter explicitly, Töpfer et al. (2020) showed that the typical malate storage capacity of C3 plants is not sufficient to allow the emergence of CAM flux distributions. Thus, changes that lead to an increased malate storage are necessary in order to develop a proper CAM pathway, these could include H^+^-ATPase (Ratajczak et al., 1994), malate channels (Pantoja and Smith, 2002), and malate/citrate antiporters (Frei et al., 2018).

So far, the proposed ODE models consider only the control of C_i_ on stomatal aperture. Although there is substantial evidence for this regulation (Cockburn et al., 1979; Males and Griffiths, 2017), we know now that it is not the only one. In order to undergo the transition from diurnal to nocturnal stomatal aperture, C3 plants need to rewire the circadian control on stomata (Hassidim et al., 2017). Comparative genomic studies have shown that genes involved in stomatal regulation have shifted their peak expression to the evening together with clock or clock-entraining genes (Yang et al., 2017; Yin et al., 2018). Thus, it is also possible that the inverse pattern of stomatal aperture was achieved before attaining a high C_i_ during the night.

Another theoretical possibility is that the circadian control on stomatal aperture was lost during CAM evolution and stomatal aperture relied mostly on the autonomous vacuolar oscillator (6). This way autonomous oscillations of malate content could drive stomatal closure during the day on their own. More research is needed to understand how autonomous oscillations in malate content and the circadian clock are integrated to control CAM rhythmicity. Currently, it is known that both, impeding the circadian input on CAM and silencing CAM, alter oscillations in CO_2_ uptake and the circadian clock itself (Boxall et al., 2017, 2020). Further ODE models integrating the clock with CAM metabolism could not only shed light on CO_2_ uptake regulation, but on the overall interplay between carbon metabolism and the clock.

An ongoing discussion is the C3-to-CAM continuum itself (Edwards, 2019; Yang et al., 2019; Schiller and Bräutigam, 2021; Winter and Smith, 2022). On one hand, the remarkable phenotypic plasticity observed in CAM plants generates a wide gradient in *δ*^13C^—an indicator of the amount of carbon fixed by CAM—in several plant lineages (Silvera et al., 2010). On the other hand, there is a clear bimodal distribution showing there are only a few intermediate phenotypes in comparison with C3 and strong CAM phenotypes, which might indicate mechanistic constraints for intermediate metabolism (Borland et al., 2011). Yang et al. (2019) argue that the continuum hypothesis underestimates the number of changes needed to achieve CAM, since available genomics data indicate at least 60 genes are involved in CAM evolution. Winter and Smith (2022) highlight that the change to nocturnal malate accumulation would involve a qualitative leap in CAM evolution since for many C3 species malate accumulates during the day.

The discussion truly appears as another instance of the old saltationism versus gradualism debate (Gould and Eldredge, 1977). However, it has opened the conversation on the pace of CAM evolution and the conditions that would facilitate either a smooth or sharp transition. Edwards (2019) analyzes the subject in terms of evolutionary accessibility and proposes there is smooth transition between C3 and weak CAM phenotypes, but for strong CAM to occur, the development of extreme succulence is necessary. Future modeling efforts on CAM metabolism could provide more evidence to discuss the subject. It would be highly informative to combine genomic information, evolutionary modeling and genome-wide models to investigate possible points of evolutionary contingency. In addition, mathematically controlled comparisons can be used to understand the evolutionary limits of the various metabolic types (Alves and Savageau, 2000; Schwacke and Voit, 2004). Another area of opportunity for CAM models is to aid in the rational implementation of CAM metabolism in C3 crops. Recently, a set of 13 CAM enzymes were overexpressed independently in Arabidopsis (Lim et al., 2019). Remarkably, the lines expressing the carboxylation module resulted in increased stomatal conductance and increased malate accumulation, and most lines from both carboxylation and decarboxylation modules had increased biomass. Feeding FBA models with experimental measurements of these plants can indicate how fluxes may be optimized after the overexpression and provide clues for novel bioengineering strategies.

Computational analysis and modeling of CAM metabolism have not only contributed to the understanding of how CAM basic components integrate for the emergence of an autonomous rhythm, and of how fluxes through central metabolism are reshaped, but may also contribute significantly to identify evolutionary selection processes. The convergence of different FBA models to CAM flux distributions provides formal mathematical evidence for low C_i_ as the main driver for the evolution of CAM. We believe that the use of these models together with evolutionary models and genomic data will shed more light on CAM evolution and assist rational engineering of CAM into C3 crops.

## Author Contributions

AB conceived the idea. AB, EM, EV, IM-C, and RA wrote the manuscript. All authors contributed to the article and approved the submitted version.

## Conflict of Interest

The authors declare that the research was conducted in the absence of any commercial or financial relationships that could be construed as a potential conflict of interest.

## Publisher’s Note

All claims expressed in this article are solely those of the authors and do not necessarily represent those of their affiliated organizations, or those of the publisher, the editors and the reviewers. Any product that may be evaluated in this article, or claim that may be made by its manufacturer, is not guaranteed or endorsed by the publisher.
